# Integrating mineralogy, geochemistry and aeromagnetic data for detecting Fe–Ti ore deposits bearing layered mafic intrusion, Akab El-Negum, Eastern Desert, Egypt

**DOI:** 10.1038/s41598-022-19760-x

**Published:** 2022-09-14

**Authors:** Sherif Kharbish, Ahmed M. Eldosouky, Omar Amer

**Affiliations:** 1 Department of Geology, Suez University, Suez, 43518 Egypt; 2 Geology Department, Faculty of Science, Zagazig University, Sharkia Governorate, Zagazig, 44519 Egypt

**Keywords:** Solid Earth sciences, Geochemistry, Geophysics, Mineralogy

## Abstract

This study delineated the Fe–Ti oxide deposit concurrencies on the layered mafic intrusion in Gabal Akab El**-**Negum (GAN), South Eastern Desert, Egypt, using aeromagnetic mapping and chemical analysis of the hosted mafic rocks and mineralogical studies. Aeromagnetic data was improved using the enhanced horizontal gradient amplitudeto detect the primary structures (edges/contacts/faults) that control the distribution of Fe–Ti ore deposit. GAN layered gabbros are differentiated into troctolite, olivine–, pyroxene–, and hornblende–gabbros. These mafic rocks primarily comprise plagioclase, olivine, pyroxene, and hornblende with Fe–Ti ores (magnetite and ilmenite). The significant variation in Mg# of clinopyroxene between 0.70 and 0.82 indicates the importance of fractional crystallization in developing layered mafic intrusion. Clinopyroxene and plagioclase thermometry yielded low temperatures similar to the fractionated primary basaltic magma. The pairs of magnetite**–**ilmenite minerals in gabbros provide equilibrium temperatures of 539.44**–**815.56, and high *fO*_*2*_, reflecting various cooling and subsolidus reequilibration phases of minerals. The enrichment of GAN gabbros in light rare–earth elements relative to heavy rare–earth elements indicates the interaction between the Fe–Ti rich mantle and the fractionated tholeiitic magmas in the back-arc setting, generating Fe–Ti oxide ores.

## Introduction

Exploring the mineral deposits of the upper crust demands integrating various geologic, structural, geophysical, datasets, geochemical, and mineralogical studies^1–4^. Recognizing geological structures is vital for investigating mineral resources and regional surveys because they can provide optimum forms for magma emplacement and fluid migration. Furthermore, it can maximize mineralization investment in various geodynamics patterns^1,5–7^. However, surface surveying might not notice considerable geologic structures that significantly assemble the mineralized arrangements and earth’s resources^8,9^.

Integration of geochemical and geophysical data could have a comprehensive application in mineral exploration. Magmatic titanomagnetite ore bodies typically exhibit complicated field evidence, they can be found as massive Fe–Ti oxides or as layers with their host rocks^10^. Therefore, aeromagnetic datasets provide the geometry of magnetized sources^1,2^ that can be associated with mineralization. Edge/contact delineation of aeromagnetic data was recently used to accurately decipher geological structures^11^.

Fe–Ti ores can be generated from two contrasting models such as a result of Fe–Ti oxide crystal sorting from magmas or accumulating of oxide melts that resulted from immiscible separation in magma^12^. In Egypt, mafic layered intrusions are frequently associated with or hosted Fe–Ti oxides^13,14^. These intrusions are scarcely exposed in the Southern Eastern Desert (SED) of Egypt, which belongs to the Arabian Nubian Shield (ANS).

**Figure 1:**
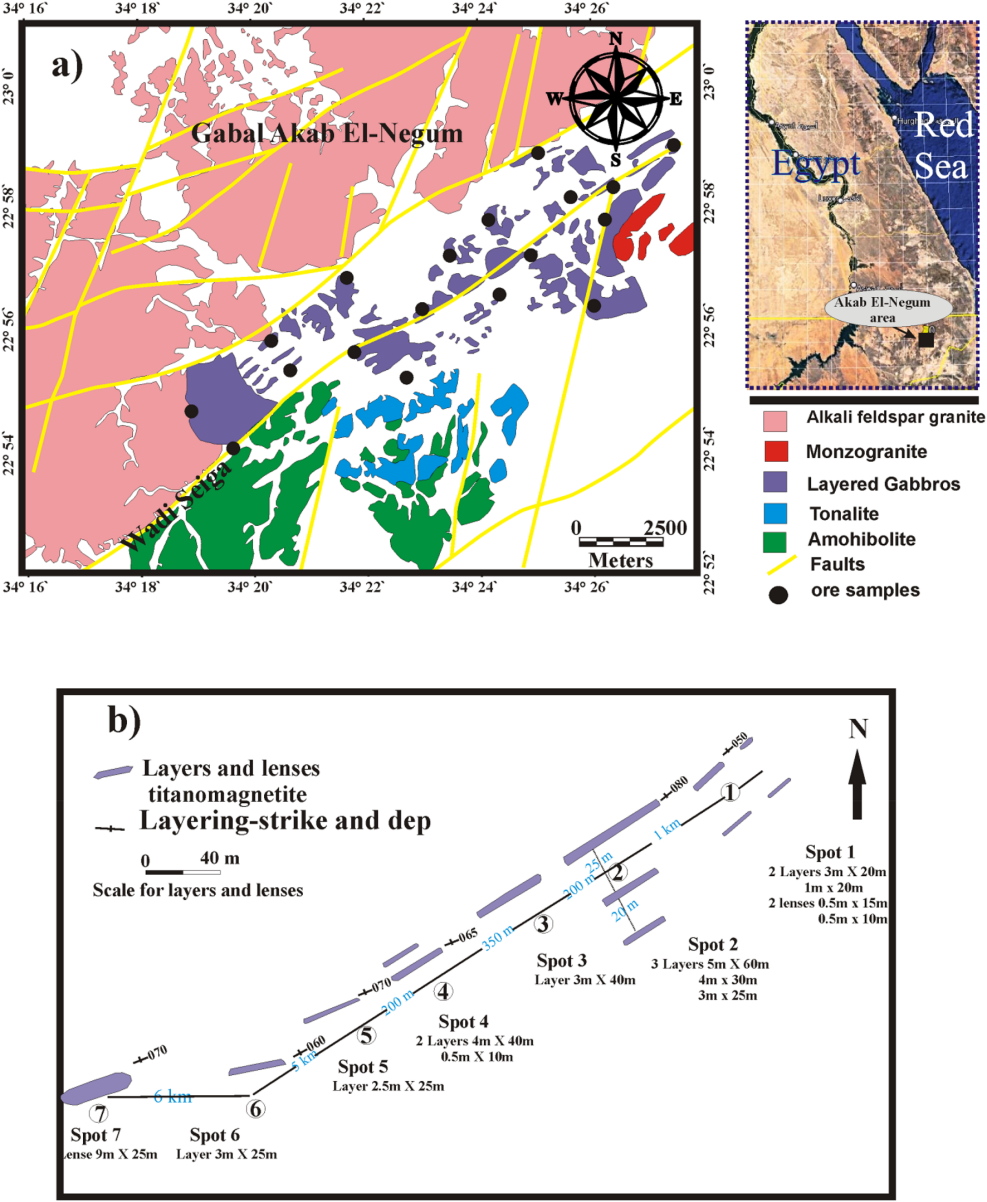
(**a**) Geological map of the study area using CorelDraw X3 on enhanced landsat8 OLI image (LC08_L1TP_173044_20211108_20211117_01_T1; USGS Earth Explorer data portal; https://earthexplorer.usgs.gov/), processed by Envi 5.4 (Trial Version; https://www.l3harrisgeospatial.com/Software-Technology/ENVI), and (**b**) Sketch of titanomagnetite ores Akab EI-Negum area, Central Eastern Desert, Egypt (Modified after Ref.^13^).

GAN intrusion (Fig. 1a) occurs as small mafic outcrops with layers ranging from centimeters to meters of layered gabbros^13^. Despite the work of^13^, the investigated area has never been studied. So far to best of our knowledge the present work aims to use an integration of both geochemistry and geophysics in order to indicate the distribution and investigate the genesis and tectonic setting of Fe–Ti oxide hosting layered gabbro of GAN area.

## Geological outlines

### Geological background

GAN area is covered by layered gabbros cutting amphibolites, monzogranite and alkali feldspar granite. This area lies between latitudes 22° 55′ to 23° 00′ N. and longitudes 34° 18′ to 34° 27′ (Fig. 1a). The intrusion's original shape has been altered to the current feature by northeast–southwest compression stress and several fault types. The layers have a NE-SW trend with sub-vertical to vertical dip. Magnetite-ilmenite ores occur as a discontinuous layer concordant with the layering of gabbros or as a disseminated type. Magnetite-ilmenite layers are 2.5 to 4 m wide and extend for about 60 m in a NE-SW direction with a NW dip direction (Fig. 1b).

### Petrography and Fe–Ti oxides mineralization

Petrographically, GAN intrusion is composed of four types of unmetamorphosed mafic rocks. (1) Troctolite is a hypidiomorphic, coarse-grained granular rock that contains cumulus plagioclase, olivine with minor amounts of clinopyroxene and hornblende. Ilmenite found as the main accessory mineral. (2) Olivine gabbro consists essentially of plagioclase, olivine with minor pyroxene. The most common secondary mineral is chlorite. Fe–Ti oxides and apatite are the main accessory minerals. The olivine is surrounded by an inner zone of orthopyroxene and an outside zone of hornblende, forming a corona texture. (3) Pyroxene gabbro consists of plagioclase and augite (Cpx), with minor hypersthene (Opx). Fe–Ti oxide minerals are found as intercumulus phases. Plagioclase forms fresh euhedral stout prisms that exhibit pericline and lamellar twinning. Sometimes plagioclase forms igneous lamination alternated with augite. (4) Hornblende gabbro composed mainly of plagioclase and hornblende with accessory apatite and opaques. Biotite and chlorite are secondary minerals. The last three gabbroic varieties are medium–coarse grained with orthocumulate texture.

**Figure 2:**
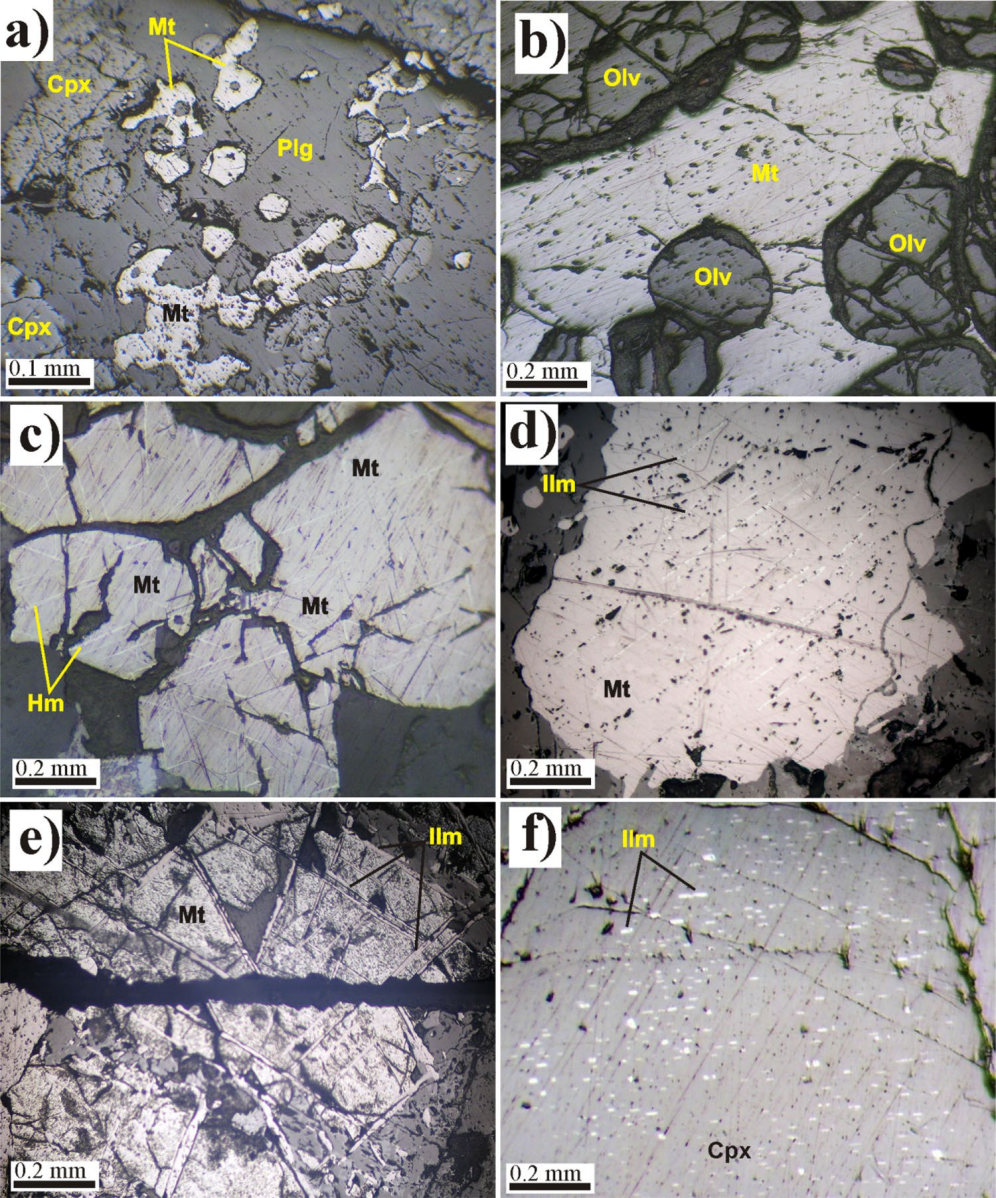
Photomicrographs of Fe–Ti oxides-rich mafic-rocks from the Akab El-Negum area. (**a**) Cumulus magnetite hosted in large plagioclase crystal, disseminated ore in pyroxene gabbro, (**b**) Intercumulus magnetite hosting cumulus olivine, semi-massive ore in pyroxene gabbro, (**c**) Magnetite slightly altered to hematite along octahedral planes, semi-massive ore in pyroxene gabbro, (**d**) Parallel ilmenite bands intergrown in large magnetite grain, semi-massive ore in olivine-gabbros, (**e**) Ilmenite bands arranged along the octahedral planes of magnetite forming trellis intergrowth, semi-massive ore in pyroxene gabbro, and (**f**) Ilmenite rods or fine needles arranged along Cpx cleavage planes, semi-massive ore in troctolite. All photos were taken under reflected light.

Fe–Ti oxides were either disseminated (5–15 vol%) or semimassive ores (20–50 vol%), represented by magnetite and ilmenite with minor hematite and goethite. They are found in cumulus and intercumulus phases. The cumulus phase is observed only in the disseminated ore in all gabbroic varieties, with the highest modal percentage in pyroxene gabbro (10–15 vol%). Fe–Ti oxide minerals form anhedral to euhedral magnetite crystals (Fig. 2a), and lesser ilmenite are hosted in silicates (e.g., plagioclase and Cpx). The intercumulus magnetiteis observed in the semimassive ore layers as space fillings and completely enclose the cumulus silicate minerals (e.g., plagioclase; Cpx and olivine; Fig. 2b). Magnetite forms subhedral to euhedral homogeneous (Fig. 2 b,c) and non-homogeneous crystals showing different exsolution textures (eg. composite, trellis, sandwich, and sandwich banded intergrowths; Fig. 2d,e). Sometimes, magnetite slightly altered to hematite along the octahedral planes forming a martitized texture (Fig. 2c). Moreover, magnetite sometimes hosts exsolved, fine isotropic rods of spinel arranged along (100) the planes^15^. Ilmeniteismainly found as intergrown bands within magnetite, large grains in contact with magnetite (composite grains), and fine needles arranged along Cpx cleavage planes forming a schiller structure (Fig. 2d,e,f). Ilmenite bands intergrown within magnetite are found as a single band forming a sandwich intergrowth, parallel bands forming banded intergrowth, or fine lamellae arranged along (111) planes of magnetite forming trellis intergrowth^16^ (Fig. 2d,e).

## Results

### Total magnetic intensity map (TMI)

**Figure 3:**
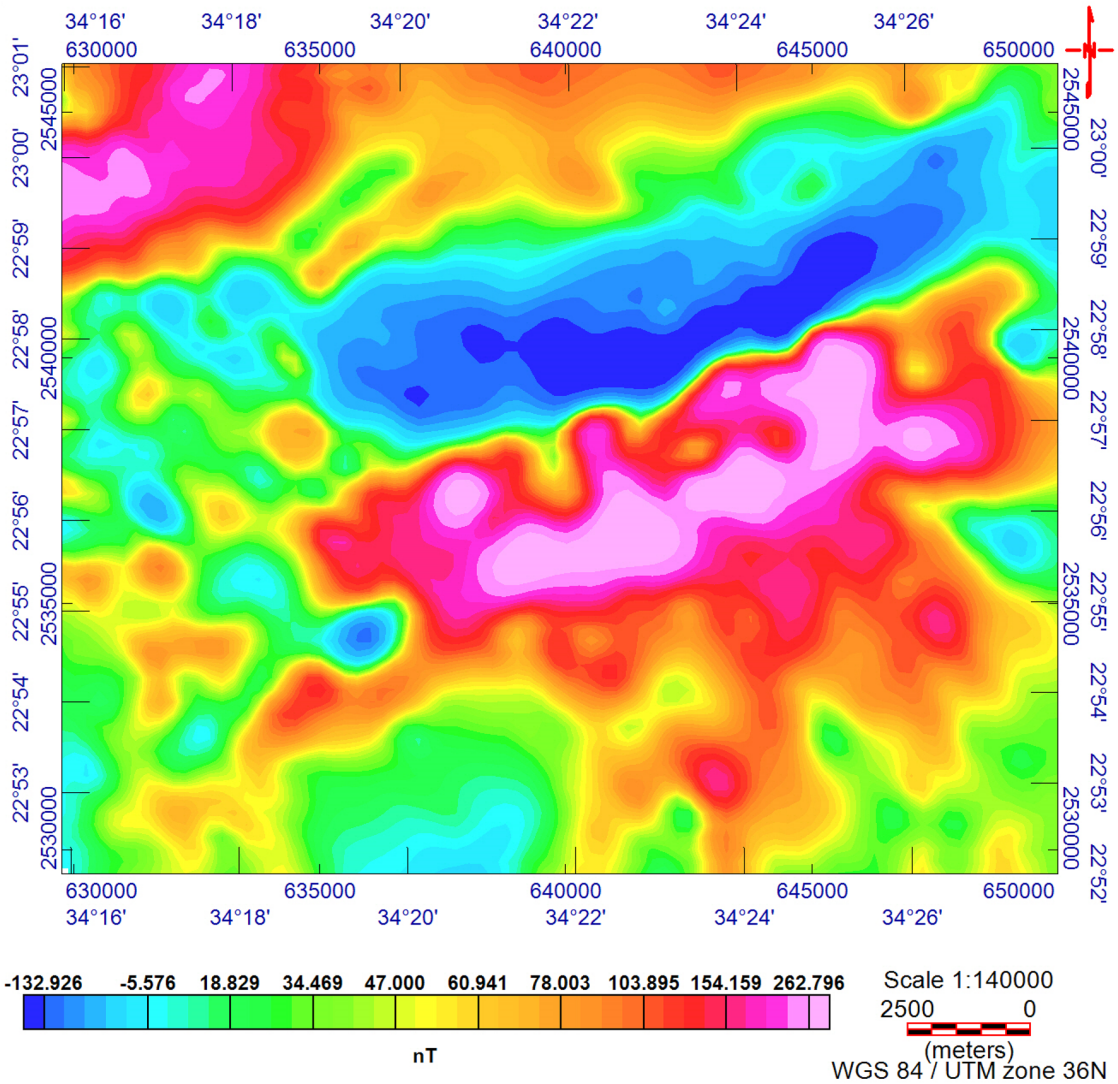
Total magnetic intensity map of the study area^55^.

**Figure 4:**
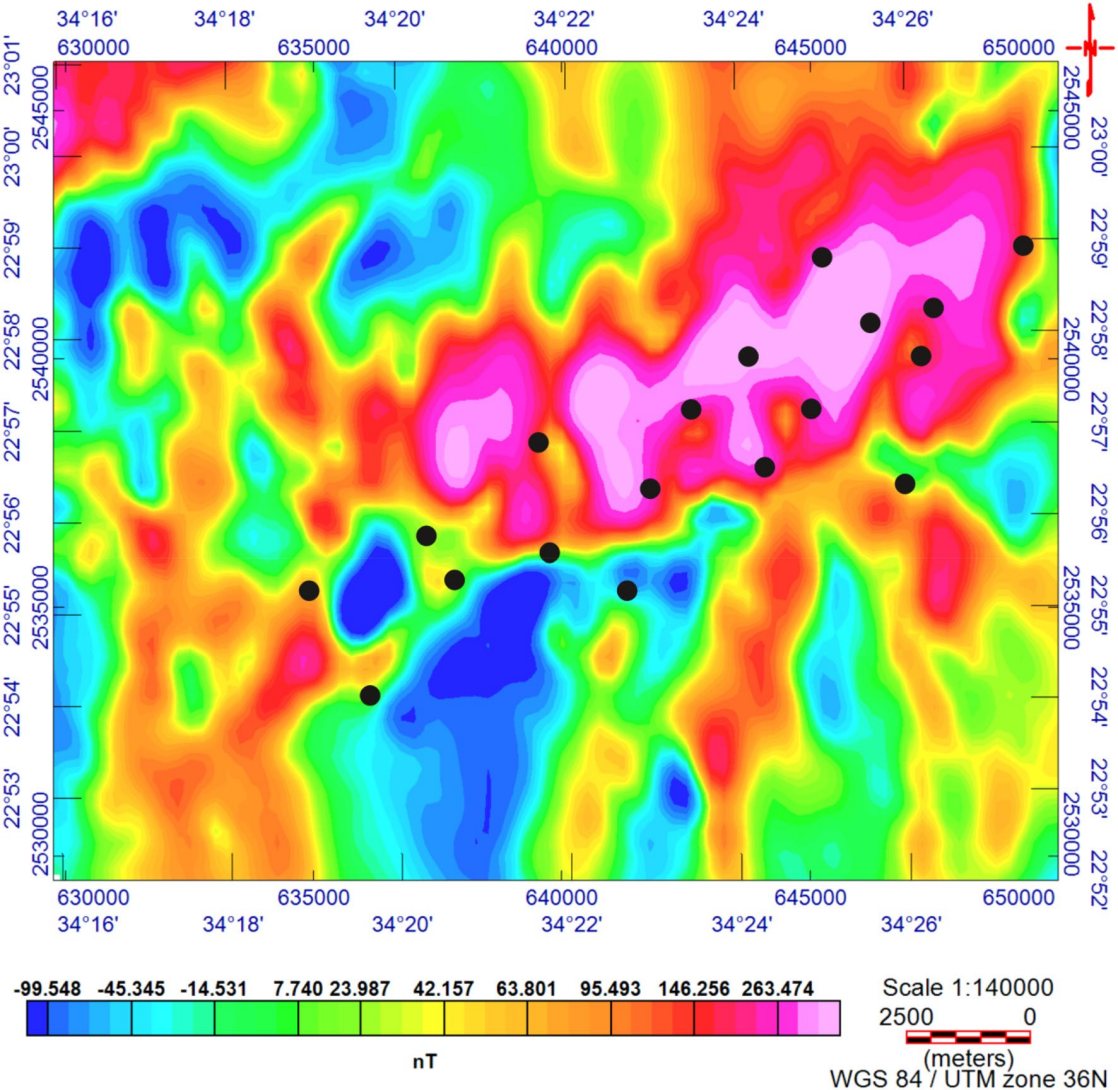
The RTP map of the Study area with rock samples.

**Figure 5:**
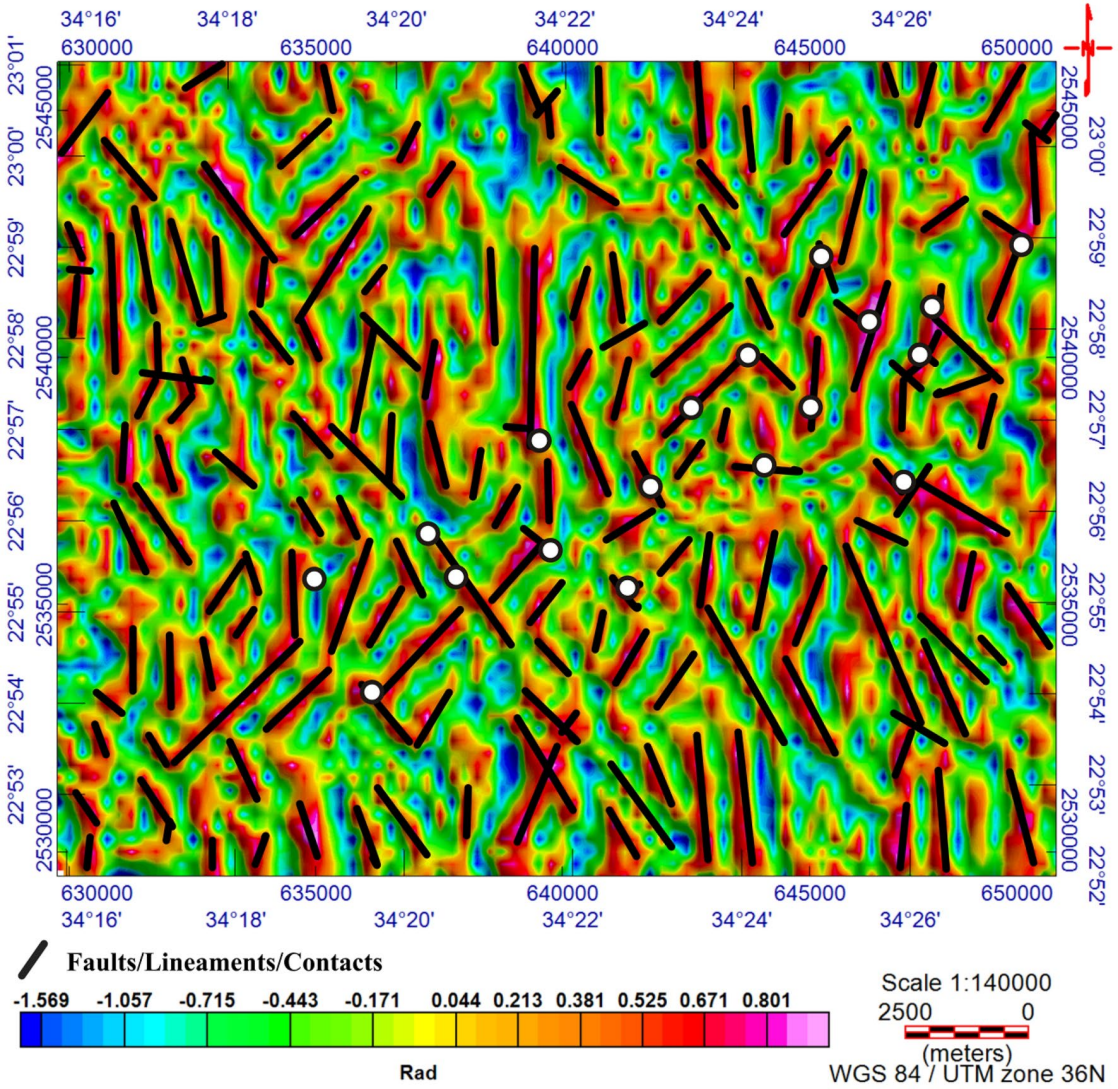
The EHGA map of the study area with rock samples.

**Figure 6:**
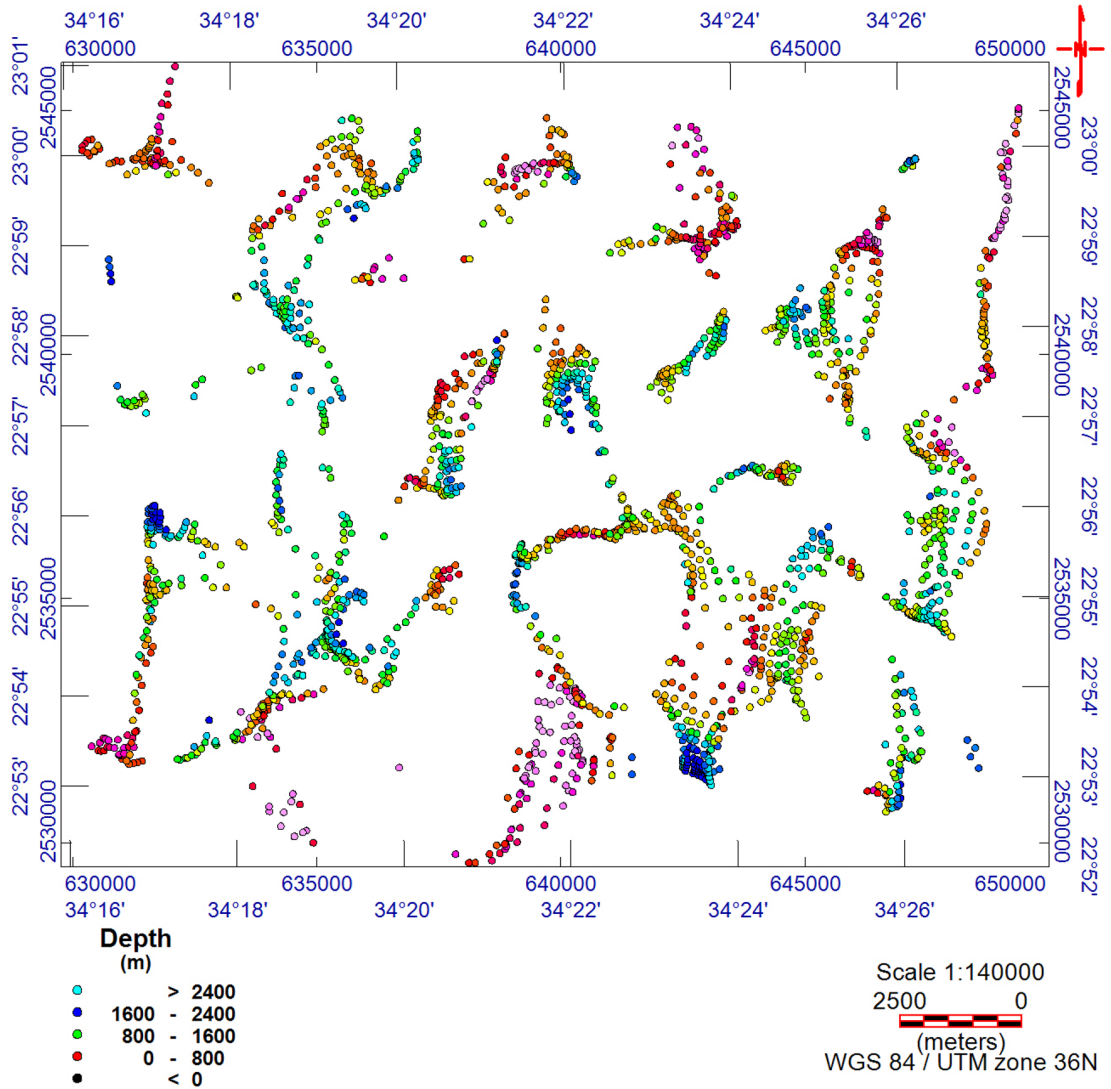
The EUD map of Akab El-Negum area.

**Figure 7:**
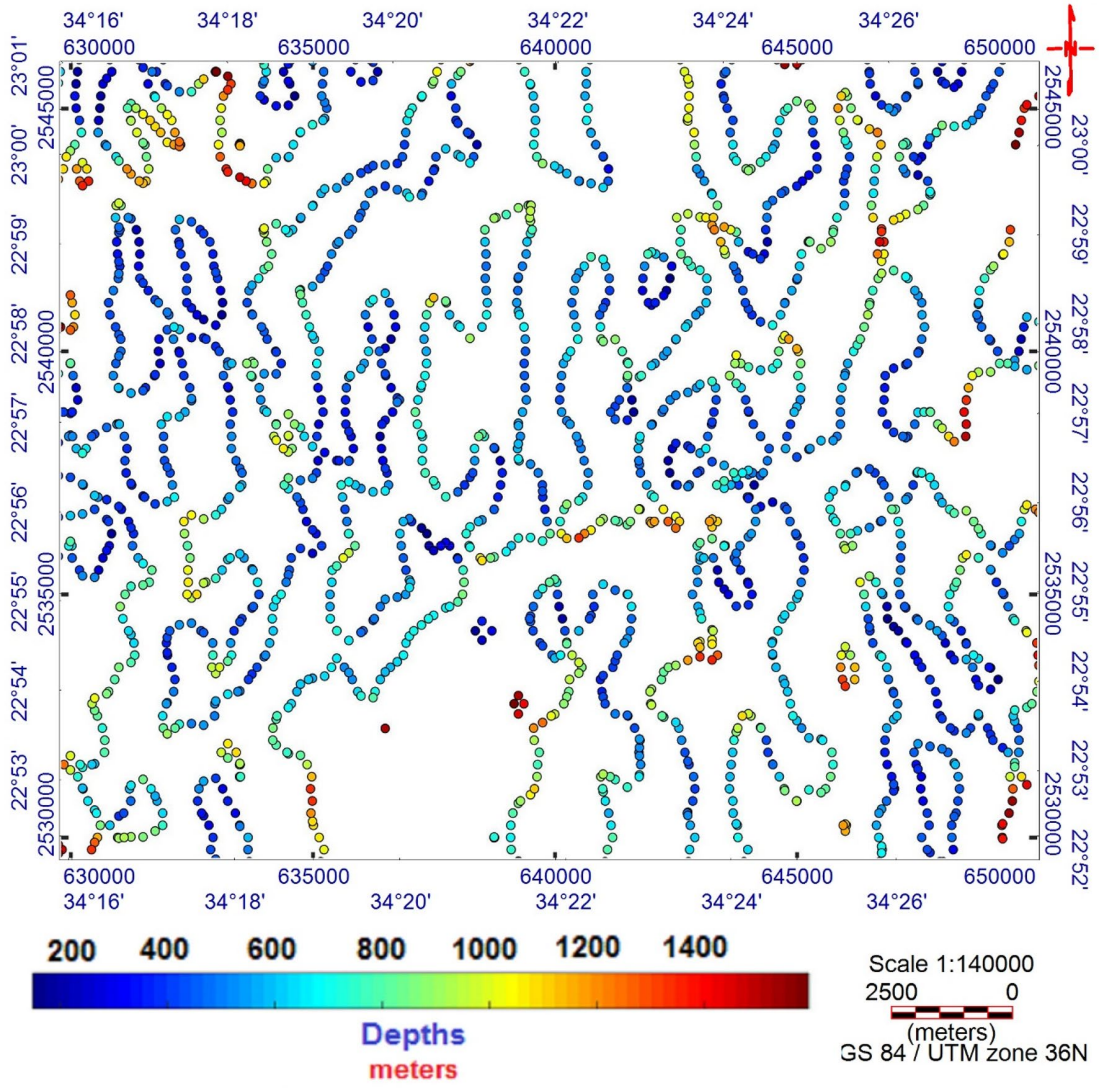
The Tilt Depth map of the magnetized ore bodies of Akab El-Negum area.

**Figure 8:**
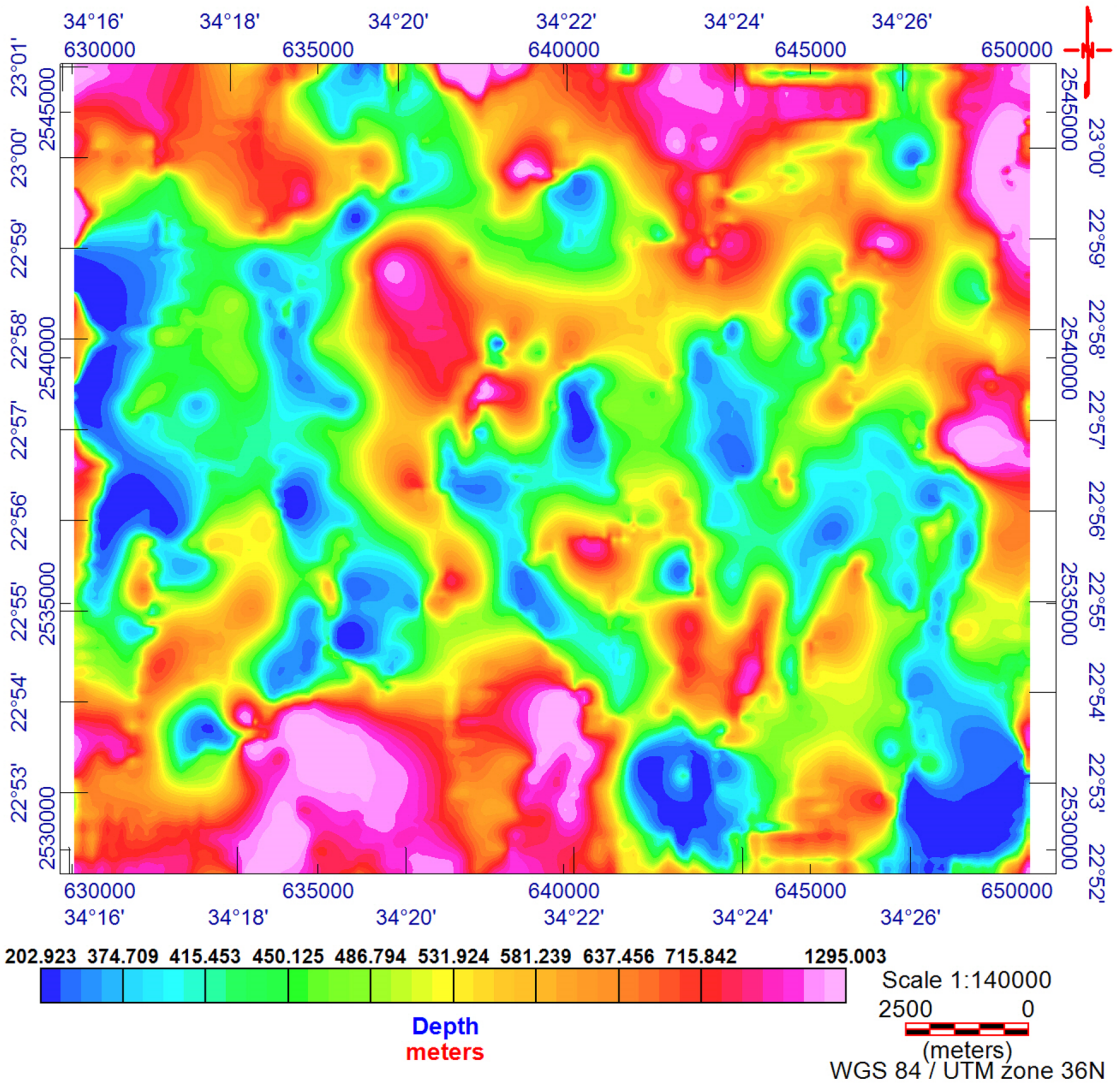
The Euler depth map of Akab El-Negum area.

The TMI map (Fig. 3) was reduced to the magnetic pole^17^ (RTP, Fig. 4). The RTP map (Fig. 4) reveals magnetic variations between—99.548 nT and > 350 nT and varied magnetic (positive and negative) anomalies. The high magnetic (positive) anomalies (red–pink colors) indicate high ferromagnetic material content within the rocks or buried magnetic bodies. The RTP map is characterized by a broad high-intensity anomaly in the northeastern part of the area ENE trending, that is dissected by N–S to NNE trends. The low magnetic (negative) anomalies appeared over the southern and northwestern parts of the area trending, N–S, NW, and NE. EHGA is applied to the study area's RTP grid. The EHGA map reflects that the dominant structures controlling of the study area are N–S, NNE, NW, NE, and NNW (Fig. 5). The EUD approach is applied to the RTP grid to detect the magnetized sources' depth and lateral extent using SI = 0.5 (Fig. 6). The result EUD map (Fig. 6) indicates that the depth of the magnetic sources varies from zero to ~ 2400 m. These sources are trend NW, N-S, NE, and ENE. Moreover, the Tilt Depth (TD) map (Fig. 7) shows the depths of ore magnetized bodies of the study area. The EUD and TD depth solutions are gridded to produce a depth map for magnetized sources (Fig. 8). The applied depth estimator in our study (Figs. 6, 7, and 8) mapped boundaries, depths, and extent of magnetized ore bodies.

### Chemistry of silicates and Fe–Ti oxide minerals

**Figure 9:**
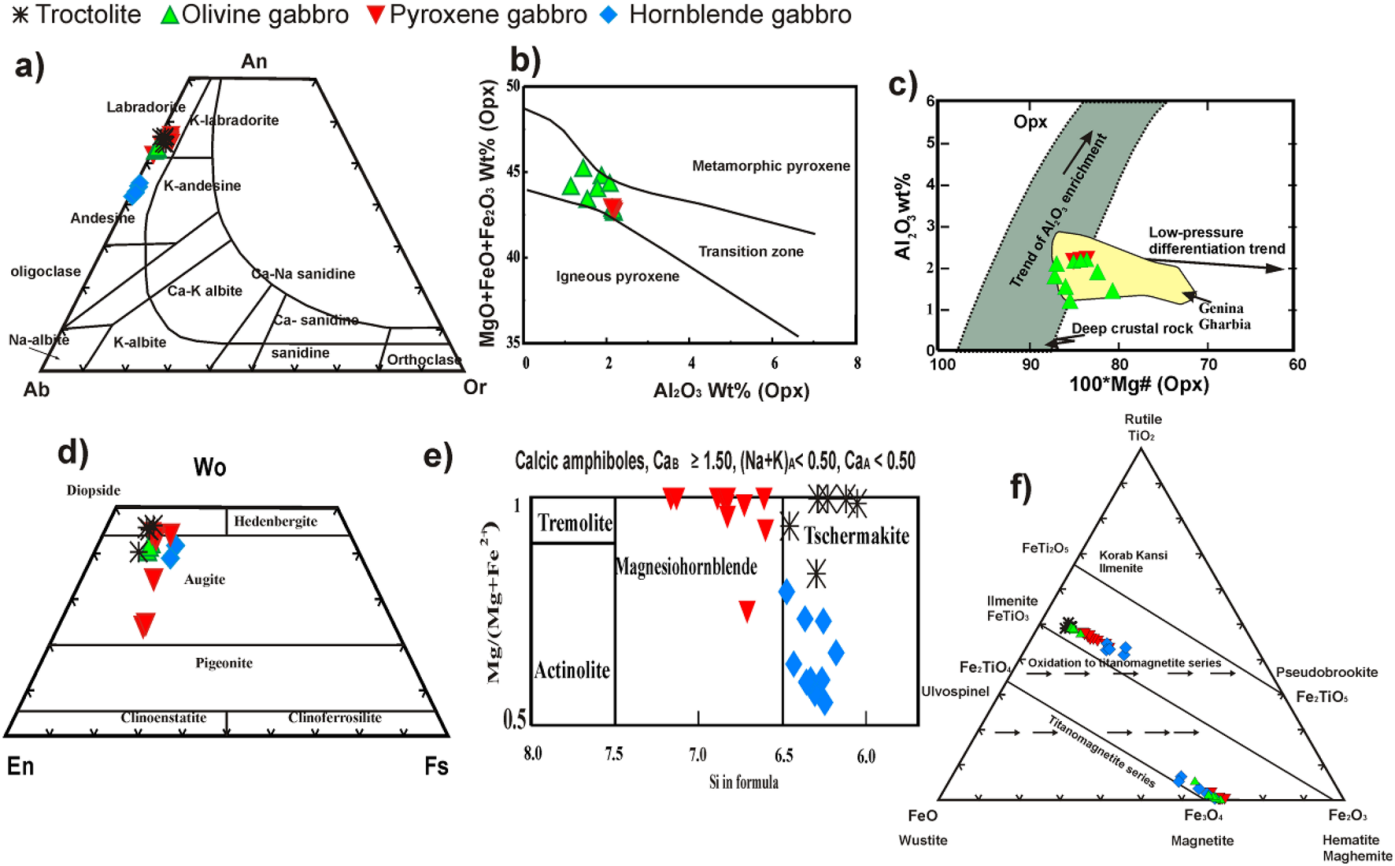
(**a**) An–Ab–Or triangular plot^18^showing the compositions of plagioclase from studied mafic rocks, (**b**) Classification of orthopyroxene^20^, (**c**) Variation diagram of Al_2_O_3_ (wt%) vs. 100*Mg# of Opxs. Fields of deep crustal rocks^21^ and Genina Gharbia Alaskan rocks^21^ are used for comparison, (**d**) Wo-En-Fs nomenclature diagram of Cpx^23^, and (**e**) Amphiboles nomenclature diagrams of^24^, and (**f**) TiO_2_–FeO–Fe_2_O_3_ classification diagram^16^.

GAN major minerals and Fe–Ti oxides analysis were listed in Table1 and Supplementary (from 1 to 6). The plagioclase composition in GAN mafic rocks (Table 1; Supplementary 1) ranges from andesine to labradorite (An_42.06–56.80_)^18^ (Fig. 9a). Andesine (An_42.06–45.20_) is recorded only in the hornblende gabbros, whereas labradorite (An_52.09–56.80_) is observed in the other varieties. Orthopyroxenes (Opxs) are observed in olivine –and pyroxene gabbros. They are of enstatite composition (En_68.95–72.08_), with a limited range of Mg# (0.81–0.87), low Cr_2_O_3_ contents (< 0.1 wt.%), and high TiO_2_ (0.22–0.36wt%; Table 1; Supplementary 2) in comparison with Opx in the ophiolitic rocks of Egypt^19^. GAN Opx plots in the transition zone between igneous and metamorphic orthopyroxenes (Fig. 9b) in the^20^ diagram. However, they follow a low–pressure differentiation pattern^21,22^ (Fig. 9c). Clinopyroxenes (Cpxs) are found in all gabbroic verities varying from augite to diopside compositions ^23^ (En_38.91–56.63_, Wo_23.87–45.44_, Fs_8.94–19.26_, Table 1; Supplementary 3; Fig. 9d) with Mg# ranging from 0.70 to 0.82. They show considerable variations in TiO_2_ (0.23–1.42 wt.%), Al_2_O_3_ (3.11–5.14 wt.%), and CaO (11.54–21.89 wt.%). GAN Amphiboles are mainly tschermakite and magnesio–hornblende^23^ (Fig. 9e). The primary amphiboles are tschermakite in composition and observed in troctolite and hornblende gabbro. Magnesio–hornblende is the main secondary amphibole (after pyroxene) and is observed only in pyroxene gabbros. The primary amphiboles (tschermakite) show higher TiO_2_ (1.46–2.21wt.%), Al_2_O_3_ (9.98–14.87 wt.%) and Na_2_O (1.24–1.78 wt.%) relative to the secondary amphiboles that replace pyroxenes (TiO_2_: 0.21–0.5 wt%, Al_2_O_3_: 3.3–5.52 wt%, Na_2_O: 0.17–1.17wt%; Table 1; Supplementary 4). Olivine is magnesian in composition, with Fo content from 69.3 to 70.19 in troctolite and 47.45 to 75.34 in olivine gabbros (Supplementary 5).

**Table 1:** Average microprobe analyses of clinopyroxene, plagioclase, orthopyroxene, hornblende, olivine and Fe–Ti oxides from Akab El-Negum mafic rocks.

Rock name	Troctolite					Olivine gabbro					
Mineral	Olv	Cpx	Plag	Hb	Ilm	Olv	Cpx	Opx	Plag	Mag	Ilm
SiO2	38.23	51.03	54.75	43.7	0.15	35.66	50.42	51.86	54.51	0.22	0.17
TiO2	0.00	1.04	0.02	1.64	48.84	0.00	0.8	0.30	0.003	1.79	47.86
Al2O3	0.00	4.7	27.26	14.02	0.02	0.21	3.17	1.82	27.96	0.65	0.02
Cr2O3	0.00	0.02		0.07	0.00	0.00	0	0.04		0.26	0.002
FeOt	26.46	6.31	0.18	11.4	48.49	35.26	7.65	16.77	0.002	84.54	48.94
MnO	0.36	0.12	0.01	0.3	1.26	0.43	0.24	0.45	0.004	0.25	1.84
MgO	34.50	15.31	0.04	12.97	0.36	27.73	16.49	26.18	0.007	0.79	0.02
CaO	0.05	20.71	5.13	11.44	0.01	0.43	20.56	0.98	10.95	0.64	0.02
Na2O	0.00	0.58	0.24	1.64		0.00	0.41	0.36	5.18		
K2O	0.00	0.02	12.05	0.39		0.00	0	0.11	0.11		
V2O3					0.03					0.34	0.26
ZnO	0.00				0.02	0.00				0.14	0.01
TOTAL	99.79	99.86	99.68	97.57	99.18	99.72	99.75	99.75	98.72	89.61	99.13
Wo		43.48					41.13	1.91			
En		44.7					45.9	71.00			
FO	69.77					58.20					
An			55.75						53.55		
Mg#	0.7	0.81		0.95	1.46	0.58	0.79	0.85		4.45	0.08

Rock name	Pyroxene gabbro						Hornblende gabbro				
Mineral	Cpx	Opx	Plag	Hb	Mag	Ilm	Cpx	Plag	Hb	Mag	Ilm
SiO2	50.8	52.99	55.33	50.12	0.10	0.11	50.98	60.89	43.87	0.32	0.13
TiO2	0.6	0.34	0.02	0.36	0.48	44.84	1.04	0.02	1.82	2.94	41.66
Al2O3	3.64	2.17	26.83	4.18	0.39	0.07	4.05	26.26	11.08	1.84	0.03
Cr2O3	0	0.05		0.25	0.24	0.02	0		0	0.35	0.02
FeOt	9.46	16.17	0.16	11.02	90.25	51.06	10.37	0.13	17.39	74.09	52.61
MnO	0.2	0.45	0.01	0.36	0.23	2.03	0.22	0.03	0.31	0.36	2.72
MgO	16.62	25.81	0.12	19.51	0.57	0.23	13.68	0.32	10.65	0.65	0.58
CaO	17.76	1.11	11.52	11.11	0.66	0.02	18.37	8.69	10.91	0.55	0.03
Na2O	0.2	0	4.94	0.78			0.99	6.09	1.49		
K2O	0.13	0	0.38	0.21			0	0.14	0.43		
V2O3					0.24	0.02				0.5	0.01
ZnO					0.18	0.01				0.02	0.02
TOTAL	99.41	99.87		97.9	93.42	98.45	99.69		97.96		
Wo	36.6	2.22					38.82	99.56			
En	47.63	72.08					40.22				
An			55.07					55.42			
Mg#	0.76	0.84		0.97	3.27	1.05	0.7		0.63	3.78	3.07

*Olv* Olivine, *Cpx* Clinopyroxene, *Plg* Plagioclase, *Hb* Hornblende, *Ilm* Iilmenite, *Mag* Magnetite, Fo = 100 * Mg/(Mg + Fe); En = Mg/(Mg + Ca + Fe + Mn); Wo = Ca/(Ca + Mg + Fe + Mn); An = Ca/(Ca + Na); Mg# = Mg/(Mg + Fe) atomic ratio.

GAN Fe–Ti minerals indicate the compositional range from near the magnetite end member (Fe_3_O_4_) to near the ilmenite end member (FeTiO_2_), similar to Fe–Ti ore deposits in Korab Kansi, SED, Egypt^19^ (Fig. 9f). Ilmenites have XIlm [Fe^2+^/(Fe^2^^+^ + Fe^3+^  + Mg + Mn)] values from 0.52 to 0.88, and contain TiO_2_ (39.86 – 49.44 wt%) and FeO^t^ (47.37–54.57 wt%) on concentrations similar to Skaergaard layered gabbros^25^ (TiO_2_ 49.15–54.73 wt%; FeO^t^ 41.36–44.27 wt%). GAN magnetites have a low TiO_2_ concentration (0.24 to 6.05 wt%) and a limited range of ulvöspinel values^26^ (0.68–18.92 mol%) (Supplement 6). FeO^t^ content varies from 66.10 to 91.54wt% due to replacement by Ti, V, Mn, and Mg, and it has low amounts of MnO (0.16–0.39 wt%), and Al_2_O_3_ (0.06–6.23 wt%) similar to magmatic magnetite of the Abu Ghalaga intrusion SED, Egypt^14^.

### Whole-rock geochemistry

GAN gabbroic rocks are fresh, as indicated by their low LOI values (0.46–2.47 wt%; Table 2). The analyzed samples show various major oxides concerning SiO_2_ content (38.05 – 49.29 wt.%; Table 2). Pyroxene gabbro (primitive rock) had a low mg# average (49.37) and high averages of Fe_2_O_3_ (19.59 wt%), TiO_2_, (1.9 wt%) Cr (223.18 ppm), Zn (117.99 ppm), and V (714.66 ppm), related to other varieties (Table 2), indicating rich Fe–Ti parental magma.GAN mafic rocks' REEs analyses revealed various REE concentrations and REE patterns (Table 2). Hornblende gabbros have the greatest REEs content (19.54–21.34, Table 2) as a result of high modal volume of hornblende, relative to coexisting silicate mineral phases^19^. This can be related directly to vary mineral assemblage due to the changes in intercumulus liquids, fractional crystallization, and the volume percentage of silicates in the modal volume.

**Table 2:** Whole-rock major (wt %), trace and rare earth elements (ppm) of Akab El-Negum mafic rocks.

Rock name	Troctolite					Olivine gabbro						
Sample No	AN15	AN16	AN17	AN18	Average	AN20	AN21	AN22	AN23	AN24	Average	
SiO2	46.21	46.17	45.89	46.19	46.12	47.98	46.66	46.71	47.32	46.93	47.12	
TiO2	0.27	0.21	0.17	0.24	0.22	0.97	1.78	1.02	1.375	1	1.23	
Al2O3	19.13	18.75	19.19	18.94	19	17.15	18.54	17.89	17.95	17.14	17.73	
Fe2O3	11.24	11.36	10.99	11.3	11.22	10.41	11.04	11.12	10.725	11.15	10.89	
MnO	0.13	0.15	0.12	0.14	0.14	0.13	0.15	0.12	0.14	0.13	0.13	
MgO	11.63	11.59	12.05	11.61	11.72	9.05	8.89	9.24	8.97	9.15	9.06	
CaO	7.91	8.04	7.89	7.975	7.95	9.89	8.44	9.43	9.165	9.66	9.32	
Na2O	2.56	2.66	2.51	2.61	2.59	2.84	2.54	2.51	2.69	2.68	2.65	
K2O	0.14	0.17	0.16	0.155	0.16	0.13	0.17	0.11	0.15	0.12	0.14	
P2O5	0.03	0.02	0.03	0.025	0.026	0.01	0.07	0.08	0.04	0.05	0.05	
LOI	0.46	0.51	0.95	0.49	0.6	1.05	1.05	1.65	1.05	1.43	1.25	
Sum	99.71	99.63	99.95	99.67	99.74	99.61	99.33	99.88	99.575	99.44	99.56	
mg#	67.21	66.9	68.48	67.06	67.41	63.27	61.47	62.21	62.35	61.92	62.24	

Rock name	Pyroxene gabbro					Hornblende gabbro						
Sample No	AN1	AN2	AN3	AN4	Average	AN30	AN31	AN32	AN33	AN34	Average	
SiO2	38.05	39.56	40.12	41.56	39.82	49.29	48.37	48.13	48.78	48.35	48.58	
TiO2	2.07	1.57	1.95	2.01	**1.9**	1.02	1.21	1.33	1.07	1.2	1.17	
Al2O3	16.88	16.08	15.84	15.37	16.04	17.2	15.9	17.42	16.53	17.51	16.91	
Fe2O3	20.17	19.75	19.11	19.31	**19.59**	8.57	10.67	8.25	10.61	8.29	9.28	
MnO	0.24	0.18	0.19	0.23	0.21	8.25	8.17	9.25	8.46	8.83	8.59	
MgO	10.42	9.05	9.42	9.71	9.65	10.21	10.32	10.09	9.81	9.95	10.08	
CaO	7.89	8.17	9.08	6.77	7.98	2.36	2.33	2.81	2.35	2.82	2.53	
Na2O	2.19	2.54	2.34	2.57	2.41	0.66	0.54	0.47	0.29	0.47	0.49	
K2O	0.14	0.14	0.17	0.12	0.14	0.2	0.19	0.21	0.13	0.21	0.19	
P2O5	0.02	0.03	0.03	0.02	0.03	0.04	0.03	0.02	0.07	0.03	0.04	
LOI	1.76	2.47	1.73	1.84	1.95	1.85	2.01	1.78	1.56	2.17	1.87	
Sum	99.83	99.54	99.98	99.51	99.72	99.65	99.74	99.76	99.674	99.83	99.73	
mg#	50.58	**47.58**	49.41	49.91	**49.37**	70.24	65.71	**70.79**	64.69	70.4	68.37	

Rock name	Troctolite			Olivine gabbro			Pyroxene gabbro			Hornblende gabbro		
Sample No	AN15	AN16	Average	AN22	AN23	average	AN1	AN2	Average	AN31	AN32	Average
As	0.66	0.73	0.7	0.6	0.68	0.64	0.56	0.95	0.76	0.85	0.8	0.83
Ba	36.49	37.45	36.97	47.59	48.25	47.92	27.58	20.22	23.9	401.25	400.11	400.68
Co	92.88	95.24	94.06	85	87.35	86.18	101.24	98.15	99.7	35.25	36	35.63
Cr	33.52	34.11	33.82	74	73.1	73.55	173.24	273.11	**223.18**	99.54	100	99.77
Cu	13.98	14.24	14.11	17.01	18.74	17.88	14.25	7.81	11.03	16.45	16.77	16.61
Ga	13.56	13.16	13.36	14	14.58	14.29	17.64	18.02	17.83	16.52	17	16.76
Hf	0.03	0.03	0.03	0.31	0.3	0.31	0.17	0.15	0.16	0.21	0.19	0.2
Li	1.85	1.97	1.91	3.44	3.54	3.49	3.68	3.76	3.72	2.65	2.85	2.75
Mo	0.07	0.08	0.08	0.07	0.07	0.07	0.09	0.08	0.09	0.09	0.08	0.09
Nb	0.3	0.28	0.29	0.3	0.29	0.3	0.43	0.39	0.41	1.42	1.39	1.41
Ni	173.55	173.25	173.4	95	93.42	94.21	112.14	109.2	110.67	88.26	89	88.63
Pb	0.5	0.47	0.49	0.42	0.36	0.39	0.38	0.37	0.38	0.41	0.39	0.04
Rb	0.2	0.21	0.21	0.3	0.34	0.32	0.52	0.63	0.58	38.95	39.2	39.08
Sc	4.1	4.05	4.08	3.63	3.78	3.71	3.74	3.24	3.49	3.89	3.75	3.82
Sr	473.3	473.24	473.27	511.02	512.06	511.54	347.15	224.12	285.64	452.19	451.13	451.66
Th	0.07	0.05	0.06	0.04	0.04	0.04	0.08	0.07	0.08	0.05	0.05	0.05
U	0.01	0.01	0.01	0.02	0.02	0.02	0.03	0.02	0.03	0.02	0.02	0.02
V	24.33	24.1	24.22	148	149.85	148.93	656.14	773.17	**714.66**	226.84	225.67	226.26
Y	1.01	0.99	1	2.21	2.17	2.19	0.73	0.68	0.71	1.25	1.33	1.29
Zn	66.1	65.81	65.96	71	69.42	70.21	124.24	111.73	**117.99**	27.54	27.01	27.28
Zr	6.77	7.34	7.06	7.95	8.09	8.02	5.78	6.71	6.25	9.25	9.75	9.5
La	1.23	1.11	1.17	1.1	1.12	1.11	0.94	1.01	0.98	8.45	8.95	8.7
Ce	2.33	2.21	2.27	2.74	2.85	2.8	2.11	2.04	2.08	5.24	6.11	5.68
Pr	0.3	0.26	0.28	0.35	0.41	0.38	0.23	0.25	0.24	0.58	0.74	0.66
Nd	1.18	1.14	1.16	1.36	1.29	1.33	0.96	0.98	0.97	2.14	2.47	2.31
Sm	0.27	0.27	0.27	0.33	0.35	0.34	0.18	0.21	0.2	1.05	1.07	1.06
Eu	0.51	0.51	0.51	0.48	0.52	0.5	0.47	0.51	0.49	0.63	0.65	0.64
Gd	0.22	0.24	0.23	0.51	0.48	0.5	0.18	0.21	0.2	0.47	0.44	0.46
ΣLREEs	6.04	5.74	5.89	6.87	7.02	6.95	5.07	5.21	5.14	18.65	20.43	19.54
Tb	0.07	0.05	0.06	0.07	0.09	0.08	0.04	0.02	0.03	0.12	0.18	0.15
Dy	0.2	0.22	0.21	0.46	0.48	0.47	0.13	0.16	0.15	0.28	0.3	0.29
Ho	0.04	0.03	0.04	0.09	0.07	0.08	0.01	0.02	0.02	0.11	0.1	0.11
Er	0.18	0.16	0.17	0.22	0.26	0.24	0.08	0.07	0.08	0.09	0.08	0.09
Tm	0.02	0.03	0.03	0.03	0.04	0.04	0.01	0.02	0.02	0.09	0.09	0.09
Yb	0.12	0.17	0.15	0.2	0.21	0.21	0.07	0.09	0.08	0.14	0.1	0.12
Lu	0.02	0.03	0.03	0.03	0.04	0.04	0.02	0.02	0.02	0.06	0.06	0.06
ΣHREEs	0.65	0.69	0.67	1.1	1.19	1.15	0.36	0.4	0.38	0.89	0.91	0.9
ΣREEs	6.69	6.43	6.56	7.97	8.21	8.09	5.43	5.61	5.52	**19.54**	**21.34**	20.44
(La/Lu)N	6.41	3.84	5.13	3.8	2.90	3.35	4.9	5.26	5.08	14.61	15.48	15.05
Eu/Eu*	6.22	6.02	6.12	3.59	3.9	3.75	7.93	7.38	7.66	2.38	2.45	2.42
(La/Yb)N	6.21	3.96	5.09	3.33	3.23	3.28	8.14	6.8	7.47	36.58	54.24	45.41
(Ba/La)	29.67	33.74	31.71	43.26	43.08	43.17	29.34	20.02	24.68	47.49	44.71	46.1
Sr/Nd	401.1	415.12	408.11	375.75	396.95	386.35	361.61	228.69	295.15	211.3	182.64	196.97

Significant values are in [bold].

**Figure 10:**
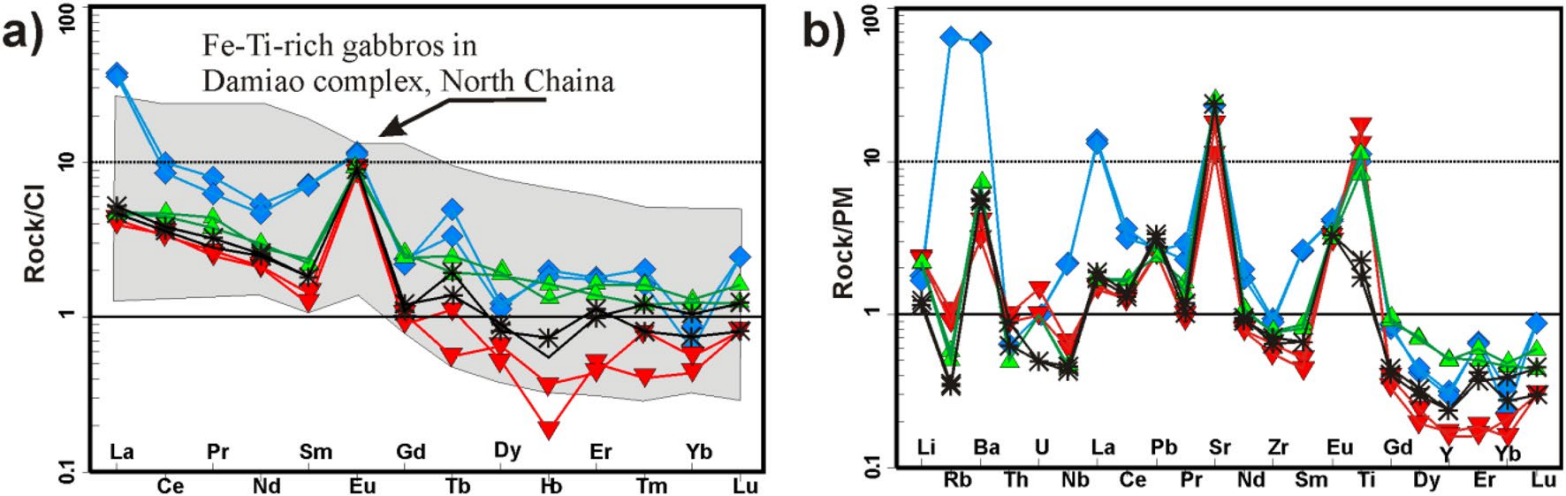
Whole-rock chemistry of Akab El-Negum mafic rocks. (**a**) Whole-rock chondrite-normalized REE patterns^27^ compared with Fe–Ti rich gabbro in Damiao complex in the North China^28^, and (**b**) Primitive mantle-normalized trace element patterns^27^.

Rock/Chondrite-normalized REE patterns of GAN mafic rocks^27^ (Fig. 10a) show enrichment in LREEs (from 5.07 to 20.43) over HRREs (from 0.36 to 1.19) and have (La/Lu)_N_ values range from 2.90 to 15.48, similar to those of the Fe–Ti rich gabbro of the Damiao complex, North China^28^. In Rock/Primitive mantle REE pattern^27^, LREEs and large-ion lithophile elements (LILEs) (Li, Sr, La, Eu, Ba, and Pb; Fig. 10b) are significantly concentrated in GAN mafic rocks compared to HREE and high field strength elements (HFSE) (Zr, Nb, Th, U), limiting chemical signatures of the subduction-zone, and adding LREE and LILE from the mantle^29^. Consequently, they effectively indicate mantle compositions that formed mafic magma^30^. The reduction in HFSE in the examined mafic rocks (Fig. 10b) indicates that the GAN intrusion was formed from a mantle source identical to back-arc basin mafics at the final spreading stage of Shikoku, Philippine^29^.

## Discussion

### Fe–Ti oxide ore deposits distribution

The RTP data (Fig. 4) show that the Fe–Ti oxide deposits are associated with high magnetic responses produced by mafic gabbros. Moreover, RTP and EHGA maps (Figs. 4 and 5, respectively) show that the Fe–Ti oxide deposits are primarily within the layered gabbros, with minor occurrences along the contact with the granites to the north and the amphibolites to the south. The correlation of collected rock samples with the RTP and EHGA data showed that the Fe–Ti oxide deposits are ENE trending and lie along the intersection zones of various fault directions. Furthermore, Figs. 5 and 6 reflect that the ENE steeply dipping and flat-lying ductile shear zones, N-S, E-W, and NW are the main tectonic frameworks controlling the study area in concordance with the N–S strike-slip shear zones^6^.

#### Pressure–temperature conditions of crystallization

**Figure 11:**
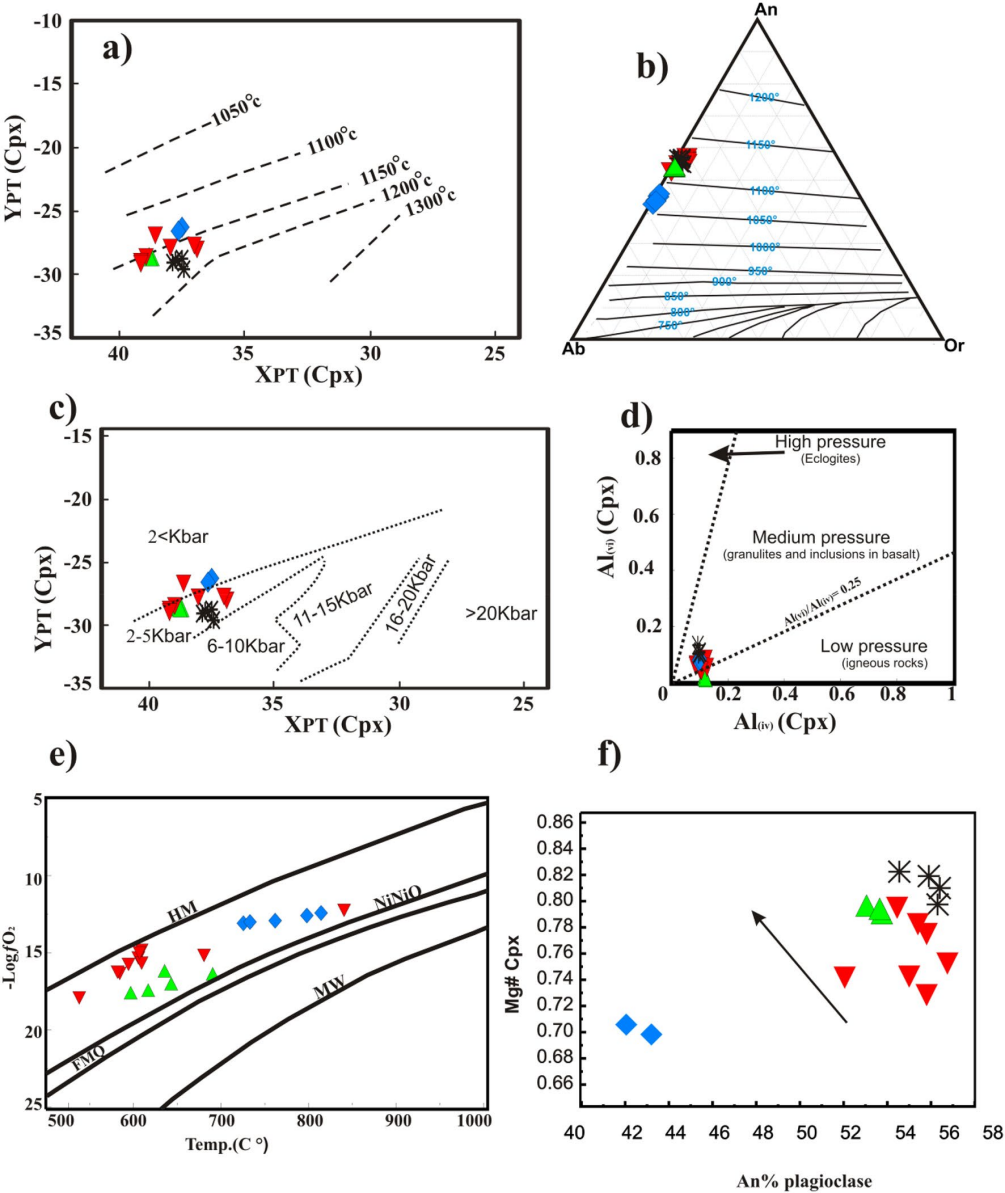
(**a**) XPT versus YPT diagram^31^ for the determination of the pyroxene crystallization temperature "XPT = 0.446SiO_2_‏ + 0.l87TiO_2_ − 0.404A1_2_O_3_‏ + 0.346FeO^t^ − 0.052MnO + ‏ 0.309MgO + ‏ 0.431CaO − 0.446Na_2_O, YPT =  − 0:369SiO_2_‏ + 0.535TiO_2_ − 0.317A1_2_O_3_ ‏ + 0.323FeO^t^ + 0.235MnO − 0.5l6MgO − 0.167CaO − 0.l53Na_2_O", (**b**) Or-Ab-An temperature diagram^32^, (**c**) XPT versus YPT pressure diagram of the pyroxene crystallization^31^, (**d**) AlVI vs. AlIV (Cpx) barometry diagram^35^, (**e**) temperature versus oxygen (ƒO2) fugacity diagram, and (**f**) An % plagioclase versus Mg# of clinopyroxene in the studied intrusion .

The clinopyroxene and plagioclase thermometers^31,32^ (Fig. 11a,b) yielded crystallization temperatures from ~ 1150 °C to 1200 °C and ~ 1050 °C to 1150 °C, respectively, close to pyroxene temperature in a fractionated basaltic magma^33^. The crystallization temperature shows higher temperature ranges for troctolite (~1200°C) than hornblende gabbro ( ~ 1050 °C), reflecting variations in magma compositions and fractional crystallization sequence near the layered intrusion temperature of Grader, Quebec, Canada ^34^( ~ 1080 °C). The crystallization pressures using XPT and YPT parameters of clinopyroxene are 2–5 kb for troctolite, pyroxene gabbro, and olivine gabbro, whereas hornblende gabbro is < 2kb^31^ (Fig. 11c). This is supported using an Al^vi^ versus Al^iv^ diagram^35^, where the analyzed clinopyroxene is plotted in medium-pressure fields for troctolite, pyroxene gabbro, and olivine gabbro and a low-pressure field for hornblende gabbro (Fig. 11d). Also, Opxs plotted in the transition zone in Fig. 9d reflect subsolidus reequilibration during cooling and magmatic crystallization under lower pressure in these rocks.

#### Equilibrium temperatures and oxygen fugacity

Equilibrium temperatures and oxygen fugacity of magnetite–ilmenite pairs^36^ were estimated using the ILMAT excel worksheet^37^. The ilmenite–magnetite pairs from olivine, pyroxene, and hornblende gabbros provide equilibration temperatures from 539.44 °C to 815.56 °C and oxygen fugacities from ΔNNO 0.68 to ΔNNO 2.13, indicating various stages of cooling history (Fig. 11e; Supplementary 6). However, their oxygen fugacity values lay between NiNiO and MH, and each group of samples follows a parallel line trend above the NNO buffer reflecting Fe–Ti oxide crystallization (Fig. 11e).

### Magmatic fractionation and contamination processes

GAN mafic intrusion has broadly mg# values from 47.58 to 70.79 (Table 2), indicating that these mafics have undergone some degree of fractional crystallization^38^. This is supported by the Cpx compositions, which exhibit gradually, decreases in Mg# from troctolite (0.81 avg.), olivine gabbro (0.79 avg.), pyroxene gabbro (0.76 avg.) to hornblende gabbro (0.71 avg.) (Supplementary 3), indicating that; the fractional crystallization occurs in parental magma^39^. The negative correlation between the An content of plagioclase and Mg# of clinopyroxene reflect the preferred Ti from the melt phase during plagioclase and pyroxene crystallization^40^ (Fig. 11f). GAN Fe–Ti rich mafic rocks are similar in Chondrite-normalized REE patterns to the Damiao complex in North China (Fig. 10a), indicating that the GAN mafic represents mixtures of cumulus minerals and trapped liquids^28^. They have positive Eu anomalies in all samples, are the weakest in hornblende gabbro and the strongest in other types, indicating plagioclase accumulation (Fig. 10a). Ilmenite and magnetite's association with primitive rocks, such as trocholite and pyroxene gabbro, indicate that fractional crystallization from Fe–Tirich parental magma had occurred^41,42^.

GAN mafic rocks show no changes in chemical and mineralogical compositions, as supported by the low LOI values < 6 (0.46–2.47 wt%; Table 2)^43^, the absence of significant Ce anomalies (Fig. 10a), unvaried Pb contents, and similar LILE distributions^44^indicate the primary geochemical features of magma with no alteration evidence. In addition, low SiO_2_ and REE contents, low Th/Nb ratios (0.04–0.23), and negative Zr anomalies, providing good evidence for the absence of crustal magma contamination through emplacement^44^.

### Genesis of Fe–Tirich magma and tectonic Setting

**Figure 12:**
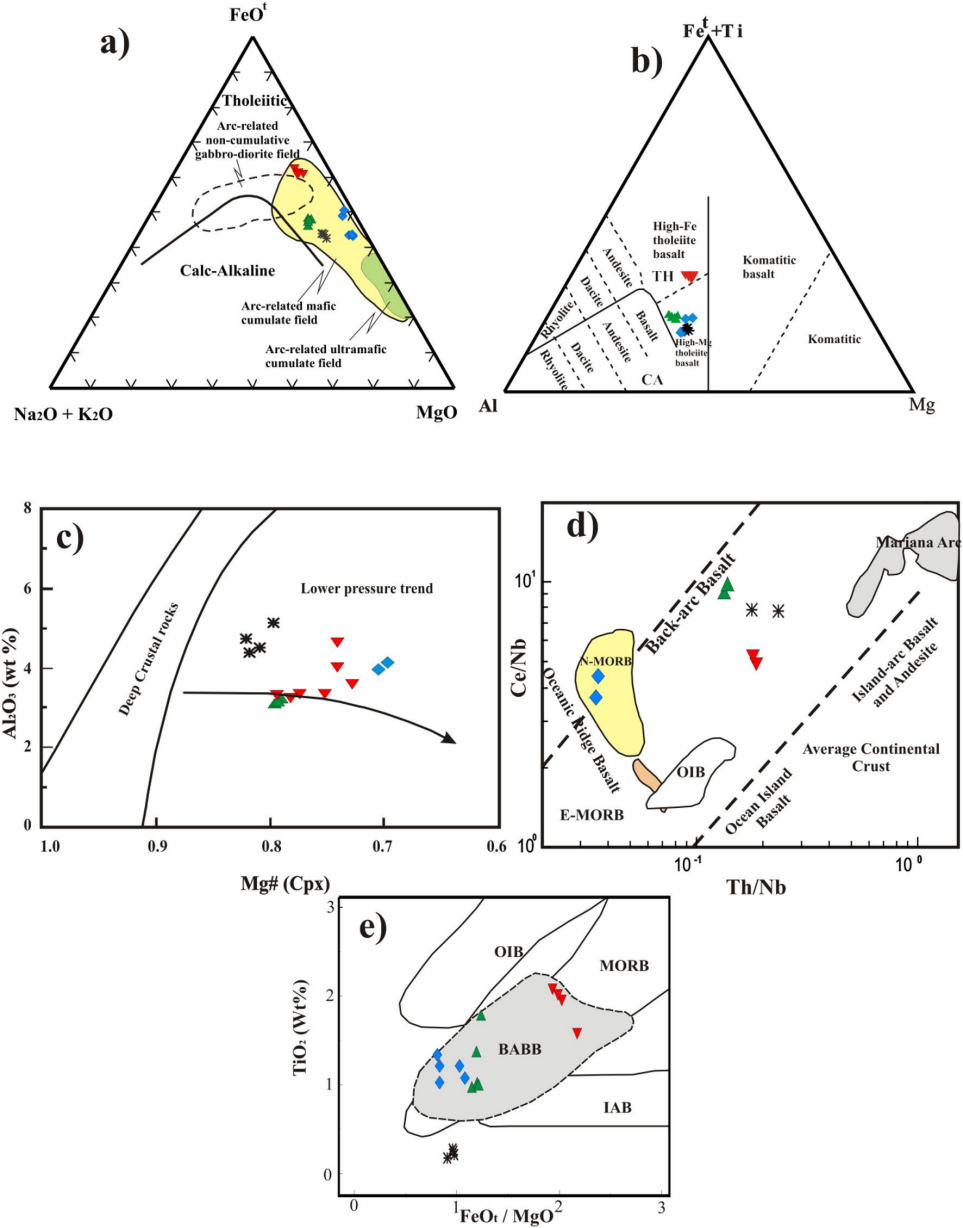
(**a**) AFM ternary diagram^47^ for the studied mafic- rocks. Fields of cumulate and non-cumulate arc-related ultramafic–mafic rocks are from^46^, (**b**) Fe^t^ + Ti–Al–Mg diagram^48^, (**c**) Variation diagram of Al_2_O_3_ (wt %) vs. 100*Mg# of clinopyroxene^21^, (**d**) Th/Nb versus Ce/Nb diagram^52^, fields are from^53^. The samples plot in the back arc field, and (**e**) FeOt/MgO-TiO2 diagram^54^.

The studied mafic rocks of GAN intrusion are good indicators to recognize the magma natures and tectonic settings of various magmatic rocks formed during its evolution. The parental magma compositions, trapped liquids, and their oxygen fugacity strongly control the accumulation of good quantities of Fe–Ti oxide ore deposits^10,19,45^. The Fe–Ti rich parental melts were produced from partially melting of Fe–Ti rich mantle sources or because of the fractionation of Fe–Ti rich mantle-derived tholeiitic magmas or a combination of both processes^10^. Based on the whole-rock chemistry, GAN mafic rocks are enriched in FeO^t^, MgO, and Na_2_O + K_2_O, similar to arc-related mafic accumulated rocks^46^ with tholeiitic affinities^47^ (Fig. 12a). A high variation in whole-rock composition can be related to the accumulation of Fe–Ti ore deposits^10^. The GAN mafic rocks have major element compositions similar to high Mg–tholeiitic basalt, except for the pyroxenegabbro, which are rich in Fe–Ti ore deposits in the high Fe–tholeiite basalt field^48^ (Fig. 12b). The GAN tholeiitic parental magma composition is the primary factor controlling the deposition of Fe–Ti ore deposits that crystallized mainly from Fe–Ti rich tholeiitic magma. Moreover, the high oxygen fugacity (ΔNNO 0.68–2.13) and the trapped liquids are crucial for controlling Fe–Ti oxide ore deposits^19,45^.

GAN mafic rocks are plotted outside the deep-level arc cumulate field and follow a low-pressure differentiation trend typical for low-pressure igneous intrusions^21^ forming in extension environment^42^ (Figs. 9c and 12c). The high difference between TiO_2_ and Na_2_O oxides^49^ indicated a great degree of partial mantle melting and aqueous fluids in the magma, leading to lower contents of incompatible-elements^49,50^. The highand uniform enrichment of LREEs/ HREEs in the studied patterns are considered a back-arc basin environment^51^. This is confirmed using the Th/Nb versus Ce/Nb tectonic discrimination diagram, where all GAN samples plot in the back-arc field, except for hornblende gabbros samples that plot in normal mid–oceanic ridge basalt (NMORB) areas^52,53^ (Fig. 12d). The FeO^t^/MgO–TiO_2_ diagram^54^ (Fig. 12e) shows that the GAN mafic intrusion is BABB except for troctolite plot out of the field. Moreover, the assemblage of arc-related mafic cumulate and MORB basalts (Fig. 12a,d, respectively) reinforces the back-arc extension environment^38^.

## Materials and methods

### Data

The aeromagnetic data of the surveyed area were collected using an Aero-Service aircraft (Cessna-Titan, Type-404), with a line separation of 1 km and 10 km tie traverse line separation at an altitude of 120 m (topography clearance). The traverse lines were instructed NE-SW with a perpendicular tie to the traverse direction^55^. The aeromagnetic data were corrected and processed by applying diurnal aircraft altitudes and removing the earth's magnetic field corrections^55^. The obtained data are in the form of total (magnetic) intensity (TMI) (Fig. 3).

### Enhanced horizontal gradient amplitude (EHGA)

^56^Presented the EHGA as:

1 $$ EHGA = {\Re }\left( {asin\left( {p\left( {\frac{{\frac{\partial HG}{{\partial z}}}}{{\sqrt {\left( {\frac{\partial HG}{{\partial x}}} \right)^{2} + \left( {\frac{\partial HG}{{\partial y}}} \right)^{2} + \left( {\frac{\partial HG}{{\partial z}}} \right)^{2} } }} - 1} \right) + 1} \right)} \right), $$

where the amplitude of the horizontal gradient (HG) is given by^57^as:

2 $$ HG = \sqrt {\left( {\frac{\partial F}{{\partial x}}} \right)^{2} + \left( {\frac{\partial F}{{\partial y}}} \right)^{2} } $$

where *p* is a constant greater than or equal to 2^56^. In our study, *p* = 3 was employed to sharply delineate the study area's edges/contacts/faults.

### Euler deconvolution (EUD)

^58^Presented the EUD as an automated method to trace the position and depth of magnetic origins for realistic magnetic data and profiles. ^59^molded it for magnetic-grid data.

The EUD method runs solution for respective or wholly structural indexes (SIs), dips, strikes, and physical properties (density or magnetization) and is generally stable. The locations and depths (× 0, y0, z0) of source bodies are calculated using the following formula:

3 $$ \frac{\delta f}{{\delta x}}\left( {x - x_{0} } \right) + \frac{\delta f}{{\delta y}}\left( {y - y_{0} } \right) + \frac{\delta f}{{\delta z}}\left( {z - z_{0} } \right) = SI\left( {B - f} \right) $$

where the observed field is ƒ at location (x, y, z). B is the field's base [regional value at (x, y, z)]. SI is the structural index^59^.

### Tilt depth (TD)

A continually operated enhancement approach for the magnetic data is the Tilt-derivative (T)^60^, which calculates the vertical-derivative amplitude of the field employing its horizontal derivatives.

4 $$ T = tan^{ - 1} \left| {\frac{{\frac{\partial f}{{\partial z}}}}{{\sqrt {\left( {\left( {\frac{\partial f}{{\partial x}}} \right)^{2} + \left( {\frac{\partial f}{{\partial y}}} \right)^{2} } \right)} }}} \right| $$

^61^explained that when the numerical formulations of the horizontal and vertical gradients over a steep contact were entered into Eq. (4), they are written as:

5 $$ TDM = tan^{ - 1} \left( {\frac{\Delta x}{{\Delta z}}} \right) $$

where ∆x and ∆z are the horizontal and vertical distances from the prevalent approximation pinpoint to the center of the boundary top.

### Sampling and chemical analysis

Depending on the aeromagnetic interpretations, the samples were systematically collected from various locations in the study area. Eighteen samples were investigated in detail [four samples from troctolite, five from olivine gabbro, four from pyroxene gabbro, and five from hornblende gabbro] (Table 1). Eight samples were investigated for trace and rare earth element (REE) analysis.

Major and trace elements were analyzed using a PW 2400 series spectrometer at Vienna University, Austria. Each powdered sample was heated to its exact weight (5 g) for 1 h at 1050 °C to determine loss on ignition (LOI). The analytical accuracy was more than 1% and 2–5%, for major and trace elements, respectively. The analytical precision and accuracy of the tested blanks, samples, and duplicates were confirmed using international standards such as African Mineral Standards (AMIS 0007). REE analysis was determined by a VG Elemental PQ3 Quadru pole inductively at the Institute of Inorganic Chemistry, Vienna University, Austria.

Mineral analyses were conducted at the Vienna University, Austria (Mineralogy and Crystallography Institute) using a Jeol JSM–6400 SEM with an EDX unit. The analytical settings were 20 eV channel width, 20 keV accelerating voltage, and cobalt as an internal gain calibration. The values of Si, K, Al, Fe, Mg, Mn, Ca, Ti, Cr, and Na were determined and calibrated on the standards: garnet, titanite, chromite, and jadeite respectively. Total of 134 spots from different minerals were studied to determine their chemical compositions (29 in plagioclase; 28 in pyroxene; 26 in amphibole; 9 in olivine and 42 in Fe–Ti oxide minerals).

## Conclusion

Our aeromagnetic dataset represents the importance of such data enhancement to map the Fe–Ti oxide deposits, which they found mainly within the layered gabbros and minor occurrences at contact with the granites and amphibolites. Furthermore, the abundance of occurrences detected primarily surrounds the strike-slip shear zones N–S.GAN magnetite and ilmenite ores are disseminated ores or layers of 2.5–4 m in width and extend approximately 60 m, concordant with the dominant aeromagnetic structures (N–S, NNE, NW, NE, and NNW) and a high intensity anomaly trending ENE (Fig. 4). These ores originated from fractionating a Fe–Ti rich basaltic magma at reequilibration temperatures from 539.44 to 815.56 °C and high fO2(ΔNNO, 0.68–2.13), indicting a variety of cooling history of ore deposits from the parental magma. Finally, GAN mafic intrusion crystallized at lower pressures and temperature (~ 1050 °C to 1200 °C), formed in a back-arc tectonic regime.

## Supplementary Information

Supplementary Information 1.

Supplementary Information 2.

Supplementary Information 3.

Supplementary Information 4.

Supplementary Information 5.

Supplementary Information 6.

Supplementary Information 7.

## Data Availability

The datasets used and/or analyzed during the current study are available from the corresponding author on reasonable request.
