# Supplementary material for: Integrating mineralogy, geochemistry and aeromagnetic data for detecting Fe–Ti ore deposits bearing layered mafic intrusion, Akab El-Negum, Eastern Desert, Egypt
Source: Sci Rep. 2022 Sep 14;12:15474. doi: 10.1038/s41598-022-19760-x (PMC9474801; doi:10.1038/s41598-022-19760-x)
Supplement: Supplementary file 1 — Supplementary Information 1. [file 41598_2022_19760_MOESM1_ESM.docx]

- Supplementary 1: Representative microprobe analyses of plagioclases in Gabal Akab El-Negum mafic rocks.
- Supplementary 2: Representative microprobe analyses of orthopyroxene in Gabal Akab El-Negum olivine gabbros and pyroxene gabbros.
- Supplementary 3: Representative microprobe analyses of clinopyroxene in Gabal Akab El-Negum mafic rocks.
- Supplementary 4: Representative microprobe analyses of amphiboles in Gabal Akab El-Negum troctolites, olivine gabbros and hornblende gabbros.
- Supplementary 5: Representative microprobe analyses of olivine in Gabal Akab El-Negum troctolites and olivine gabbros.
- Supplementary 6: Representative microprobe analyses of Fe-Ti oxides in Gabal Akab El-Negum mafic rocks.
